# Draft genome sequence of an invasive plant *Lantana camara L.*

**DOI:** 10.6026/97320630018739

**Published:** 2022-09-30

**Authors:** Adwait G. Joshi, P. Praveen, Uma Ramakrishnan, Ramanathan Sowdhamini

**Affiliations:** 1 National Centre for Biological Sciences (NCBS-TIFR), GKVK campus, Bangalore 560065, India; 2 Molecular Biophysics Unit, Indian Institute of Science, Bengaluru 560012, Karnataka, India; 3 Institute of Bioinformatics and Applied Biotechnology, Biotech Park, GN Ramachandran Road, Electronics City Phase 1, Bengaluru 560100, Karnataka, India

## Abstract

*Lantana camara* L. is an invasive species of global concern. An ornamental plant originating from central America, it has now spread across natural and human-dominated habitats across tropical and subtropical regions globally. Understanding the population and evolutionary genetics of this species could help gain deeper insights into invasion biology, and provide tools for more effective management. Such investigation would require a relatively good quality genome assembly. While there have been reports of a transcriptome, it has been challenging to construct the genome assembly because of the large genome size. We present here the first draft genome assembly of *Lantana camara* L. which has an N50 value of 62 Kb, genome completeness of 99.3% and genome coverage of 74.3%. We hope that such an assembly will help researchers study colonization history, the genetic basis of adaptation and invasiveness, and help design strategies to contain the invasiveness of this plant, allowing biodiversity recovery in several parts of the globe.

## Background:

*Lantana camara* L. (Verbenaceae) is a perennial shrub, originating from central America. Although originally introduced to different parts of the world by European travelers as an ornamental plant, it soon became an invasive species in many non-native tropical and sub-tropical areas. The impact of invasive species is a serious concern globally and lantana has been regarded as one of ten worst invasive species in the world. In India, the invasion in native forests has been associated with decline in wildlife habitats and native plants [1, 2]. There are several cultivars of this plant and the plant exhibits polyploidy making it difficult to manage the invasiveness of lantana. There have been transcriptomic studies on this plant where they have focused on the candidate genes involved in unreduced gamete formation, stress response [3] and secondary metabolite production [4]. Recently, genome size and chromosome number of five different lantana species was studied [5]. The study alluded to the complexity in the context of genomic content and the necessity of genomic data and its utility in studying the gene composition. We present here the first draft genome assembly of *Lantana camara*, which will be useful for identifying the gene composition and studying genomics properties in reference to its adaptation, colonization history and invasiveness.

## Methodology of development:

The leaves of a diploid *Lantana camara* plant were collected from the National Centre for Biological Sciences, in the University of Agricultural Sciences, Bangalore (Figure 1-A - see PDF). DNA extraction and the subsequent sequencing using 10X Chromium sequencing technology was conducted by AgriGenome Labs Pvt. Ltd. The library for sequencing was prepared using Chromium Genome Reagent Kit and the DNA quality was tested using Qubit and Tapestation. The genome size was estimated to be 2538 Mb (2C: 2.59 pg) based on DNA content calculation using flow cytometry. The sequencing data contained 505,154,448 paired-end reads (76,234.40 Mb of data). These reads were processed through quality control checks using Trimmomatic (v0.39) (Figure 1-B - see PDF) [6]. The read quality assessment before and after trimming was carried out using FASTQC [7]. The effective read length was set at a threshold of 140 bp and a phred score cutoff of 15 was used. The Supernova assembler (v2.1.1) was used to assemble these reads [8]. The 1,887,308,127 bp assembly was constructed with an N50 value of 62,974 bp which covered 74.35% of the genome (Table 1 - Assembly statistics - see PDF). This draft genome assembly was evaluated through the QUAST (v5.0.2) tool [9]. The longest scaffold was 4,355,265 bp in size, while there were 26,057 scaffolds with length greater than 10 Kb. The genome completeness assessed using BUSCO revealed that the genome is 99.3% complete considering the complete (96.5%) and fragmented (2.8%) reference genes (Figure 1-C - see PDF) [10].

## Utility to the biological community:

We aim to annotate this assembly and study the gene content in this plant. The genomic features responsible for its demographic success could be investigated in the context of specific phenotypes that may be driven by genomic adaptations like transcription factors, chemical adaptations like secondary metabolites, and physiological adaptations such as stress tolerance. The genome can provide a platform to perform comparative genomics with other varieties and related plants. The genome can also be useful while pursuing transcriptome based analysis for this plant under several conditions. *Lantana camara* is known for its polyploidy which is attributed to the phenomenon like unreduced gamete formation. The transcriptome has been used earlier to investigate this phenomenon [3]. It is possible that this draft genome assembly can be used to understand more about mechanisms that enable extensive polyploidy observed in the lantana group of plants. The current draft assembly of *Lantana camara* is the first reported version of its genome. However, we caution that, based on the estimated genome size, this assembly covers only 74.35% of the genome. Although the N50 value is 62,974 bp, the NG50 value is a little above 13 Kb considering the genome size of 2.6 Gb along with LG50 value of 17,464 contigs (Figure 2 - see PDF). Therefore, there is scope for improving the assembly and its coverage.

## Future developments:

We hope to use the current version of the assembly for annotation and predicting the gene models and the repeat content. A targeted approach, where we investigate how lantana is able to survive in such arid, drought-prone habitats could be more instructive. Further, several secondary metabolites could be responsible for creating allelopathic responses and could inhibit the growth of other (mostly native) plants, giving lantana a competitive edge [11]. We will use this genome assembly to identify candidate genes of such enzymes synthesizing these metabolites. Using the current genome assembly and a large number of samples collected across India, we are trying to answer fundamental questions such as the types of lantana present and its invasion history. There are different phenotypic variants of invasive lantana in India. Currently, we are doing a ddRADseq-based study to understand the genetic differences between these variants and their taxonomic status. Using this data, we are also trying to trace the invasion history of lantana in India. Finally, we also aim to improve the assembly by using sequencing data obtained from other sequencing platforms, allowing better genome coverage over the current assembly.

## Data availability:

The sequencing reads raw data has been uploaded on NCBI SRA (SRR21120206) linked with the BioProject entry (PRJNA861093). The genome assembly is available on request.

## Author's contribution:

PP collected the tissue for DNA extraction and carried out the genome size estimation. AGJ performed the sequencing data analysis and assembly. AGJ wrote the initial draft of the manuscript with inputs from all the authors. All authors read and approved the manuscript. RS and UR conceived and designed the project.
